# Crystal structure of tris­[4-(di­methyl­amino)­pyridinium] tris­(oxalato-κ^2^ *O*,*O*′)chromate(III) tetra­hydrate

**DOI:** 10.1107/S2056989015020113

**Published:** 2015-10-31

**Authors:** Noé Makon ma Houga, Frédéric Capet, Justin Nenwa, Gouet Bebga, Michel Foulon

**Affiliations:** a Department of Inorganic Chemistry, University of Yaounde 1, POB 812 Yaounde, Cameroon; b Unité de Catalyse et de Chimie du Solide, UMR 8181, Ecole Nationale Supérieure de Chimie de Lille, Université Lille1, 59650 Villeneuve d’Ascq Cedex, France; c Higher Teacher Training College, Chemistry Department, University of Yaounde 1, POB 47, Yaounde, Cameroon; d UFR de Physique, Université Lille1, 59650 Villeneuve d’Ascq Cedex, France

**Keywords:** crystal structure, 4-(di­methyl­amino)­pyridine, tris­(oxalato)chromate(III), hybrid salt, hydrogen bonding, π–π inter­actions

## Abstract

In the title hybrid salt, (C_7_H_11_N_2_)_3_[Cr(C_2_O_4_)_3_]·4H_2_O, the central Cr^III^ ion of the complex anion (point group symmetry 2) is coordinated by six O atoms from three chelating oxalate(2−) ligands in a slightly distorted octa­hedral coordination sphere. The Cr—O bond lengths vary from 1.9577 (11) to 1.9804 (11) Å, while the chelate O—Cr—O angles range from 82.11 (6) to 93.41 (5)°. The 4-(di­methyl­amino)­pyridinium cations (one situated in a general position and one on a twofold rotation axis) are protonated at the pyridine N atoms. In the crystal, N—H⋯O and O—H⋯O hydrogen bonds link the cations and anions into a three-dimensional network. π–π inter­actions between the pyridine rings of adjacent cations provide additional stabilization of the crystal packing, with the closest centroid-to-centroid distances being 3.541 (1) and 3.575 (1) Å.

## Abstract 2

In the crystal structure of the title compound, the mol­ecular components, *viz.* 4-(di­methyl­amino)­pyridinium cations, tris­[oxalatochromate(III)] anions and lattice water mol­ecules, are linked through O—H⋯O and N—H⋯O hydrogen bonds into a three-dimensional network. Additional π–π inter­actions between pyridinium rings stabilize this arrangement.

## Chemical context   

The coordination chemistry of oxalate (C_2_O_4_^2−^) continues to receive considerable attention because of the ability of this ion to act as a remarkably flexible ligand system in complexations with a wide range of metal ions (Martin *et al.*, 2007 ▸). Over the last decade, Bélombé and coworkers (Bélombé *et al.*, 2003 ▸) prepared a novel barium-oxalatochromate(III), {Ba_6_(H_2_O)_17_[Cr(ox)_3_]_4_}·7H_2_O, and demonstrated the use of this complex as a suitable precursor for the synthesis of multi-functional crystalline materials (Bélombé *et al.*, 2009**a* ▸,b* ▸; Mbiangué *et al.*, 2012 ▸). Moreover, this complex has received much attention in the field of materials science for its use as a convenient route for the preparation of technologically important metallic composite oxides (Neo *et al.*, 2006 ▸). As part of our ongoing research program, we have now combined this versatile barium-oxalatochromate(III) complex with 4-(di­methyl­amino)­pyridinium oxalate to isolate the organic–inorganic hybrid salt, (C_7_H_11_N_2_)_3_[Cr(C_2_O_4_)_3_]·4H_2_O.
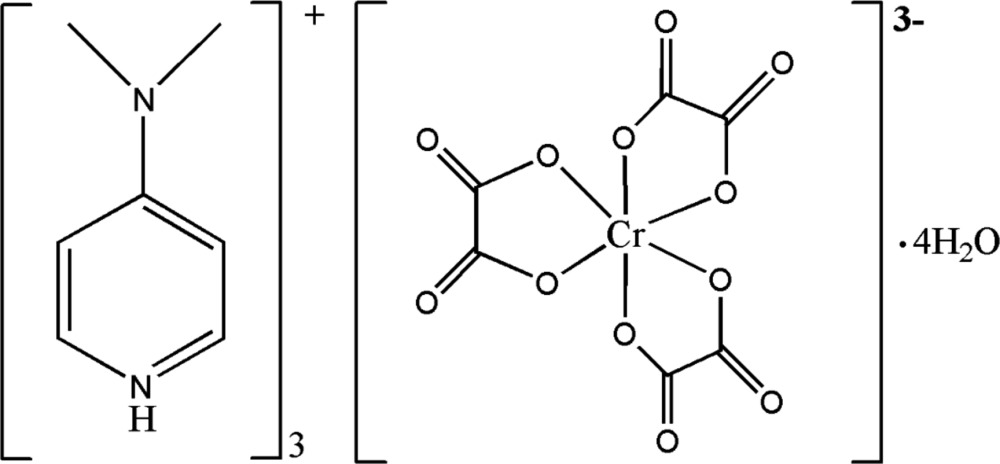


## Structural commentary   

**Figure 1:**
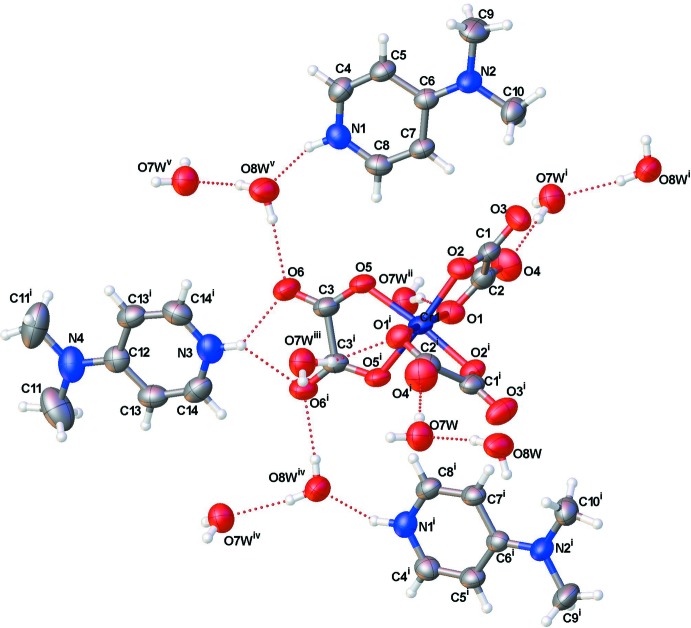
The mol­ecular components of the title compound. Displacement ellipsoids are drawn at the 50% probability level. Hydrogen bonds are shown as dashed lines. [Symmetry codes: (i) 1 − *x*, *y*, 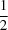 − *z*; (ii) −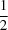 + *x*, 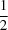 − *y*, −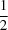 + *z*; (iii) 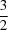 − *x*, 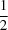 − *y*, 1 − *z*; (iv) 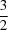 − *x*, −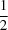 + *y*, 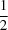 − *z*; (v) −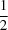 + *x*, −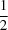 + *y*, *z*.]

The mol­ecular components of the title compound are shown in Fig. 1 ▸. The asymmetric unit contains one and a half 4-(di­methyl­amino)­pyridinium cations, one half of the tris(oxalato)chromate(III) complex anion and two lattice water mol­ecules. The entities are completed by application of twofold rotation symmetry. The central Cr^III^ ion of the complex anion is coordinated by six O atoms from three chelating oxalato(2−) ligands in a slightly distorted (2 + 2 + 2) octa­hedral coordination sphere. The chelate O—Cr—O angles range from 82.11 (6) to 93.41 (5)°. The Cr—O bond lengths vary from 1.9577 (11) to 1.9804 (11) Å and are similar to those found in the guanidinium tris­(oxalato)chromate(III) salt (Golič & Bulc, 1988 ▸). Bond lengths and angles in the organic cations, [C_7_H_11_N_2_]^+^, are in agreement with those found in salts with the same cationic entity (Nenwa *et al.*, 2010 ▸; Ghouili *et al.*, 2010 ▸; Benslimane *et al.*, 2012 ▸; Ben Nasr *et al.*, 2015 ▸).

## Supra­molecular features   

**Figure 2:**
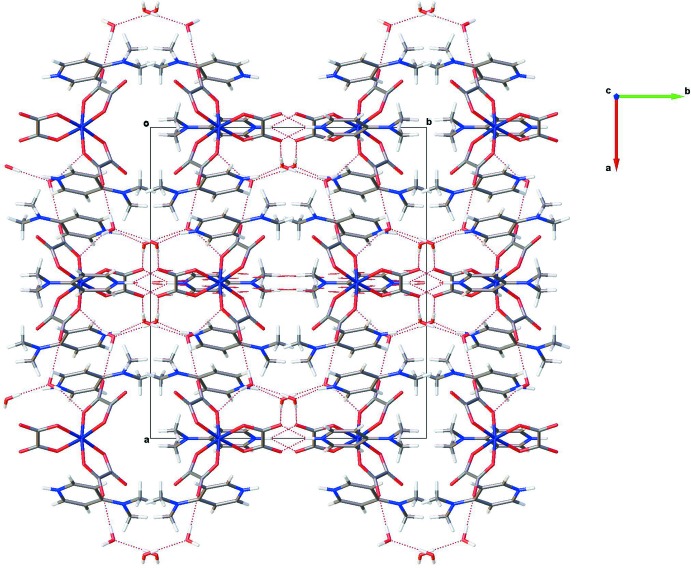
Projection on the *ab* plane of the crystal structure of the title compound. Hydrogen bonds are shown as dashed lines.

**Figure 3:**
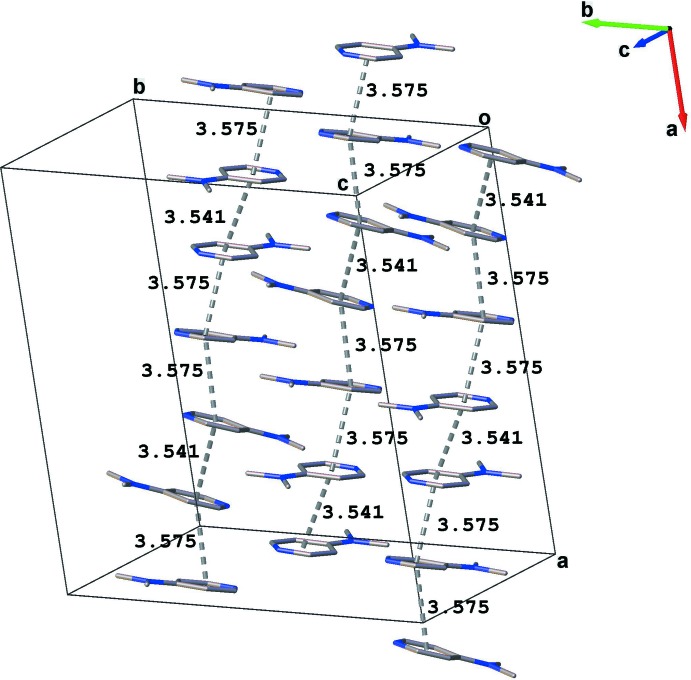
π–π stacking inter­actions (dashed lines) between adjacent organic cations in the title compound.

In the title compound, the crystal packing is stabilized by a network of inter­molecular N—H⋯O and O—H⋯O hydrogen bonds linking the coordination octa­hedra, 4-(di­methyl­amino)­pyridinium cations and lattice water mol­ecules (Table 1 ▸, Fig. 2 ▸). In addition, π–π stacking inter­actions [centroid-to-centroid distances of 3.541 (1) and 3.575 (1) Å] between the pyridine rings contribute to the stabilization of the three-dimensional network (Fig. 3 ▸).

## Synthesis and crystallization   

The title compound was obtained by reaction of an aqueous solution of the freshly prepared barium-oxalatochromate(III) salt {Ba_6_(H_2_O)_17_[Cr(C_2_O_4_)_3_]_4_}·7H_2_O (1 mmol, 2.536 g), with an aqueous solution of 4-(di­methyl­amino)­pyridine (12 mmol, 1.464 g) and oxalic acid (6 mmol, 0.756 g). The mixture was stirred at 333 K for about 30 minutes and then cooled to room temperature and filtered. The title compound crystallized by slow evaporation of the solvent at room temperature in form of light-violet crystals with dimensions up to 3 mm within a few weeks.

## Refinement   

Crystal data, data collection and structure refinement details are summarized in Table 2 ▸. H atoms bonded to C atoms were positioned geometrically and allowed to ride on their parent atoms with C—H = 0.93 Å and *U*_iso_(H) = 1.2*U*_eq_(C) for aromatic and 0.96 Å and *U*_iso_(H) = 1.5*U*_eq_(C) for methyl H atoms. H atoms of water mol­ecules as well as those bonded to N atoms were located from a difference Fourier map. Water H atoms were refined with soft restraints on O—H and H⋯H distances [O—H = 0.82 (1) Å and H⋯H = 1.30 (2) Å] and *U*_iso_(H) = 1.5*U*_eq_(O) whereas H atoms bonded to N atoms were refined freely.

## Supplementary Material

Crystal structure: contains datablock(s) I. DOI: 10.1107/S2056989015020113/wm5230sup1.cif

Structure factors: contains datablock(s) I. DOI: 10.1107/S2056989015020113/wm5230Isup2.hkl

CCDC reference: 1400490

Additional supporting information:  crystallographic information; 3D view; checkCIF report

## Figures and Tables

**Table 1:** Hydrogen-bond geometry (, )

*D*H*A*	*D*H	H*A*	*D* *A*	*D*H*A*
N1H1O8*W* ^i^	0.91(2)	1.84(2)	2.702(2)	157.8(19)
N3H3O6^ii^	0.92(4)	2.12(3)	2.879(3)	139(1)
N3H3O6^iii^	0.92(4)	2.12(3)	2.879(3)	139(1)
O7*W*H7*WA*O4^iv^	0.83(1)	1.99(1)	2.819(2)	178(3)
O7*W*H7*WB*O1^v^	0.82(1)	2.12(1)	2.9079(19)	161(3)
O8*W*H8*WA*O7*W*	0.81(1)	1.95(1)	2.7578(19)	172(3)
O8*W*H8*WB*O6^vi^	0.82(1)	1.99(1)	2.8007(19)	175(3)

Symmetry codes: (i) 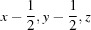; (ii) 
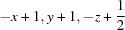
; (iii) 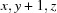; (iv) 
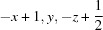
; (v) 
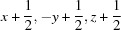
; (vi) 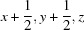.

**Table 2:** Experimental details

Crystal data	
Chemical formula	(C_7_H_11_N_2_)_3_[Cr(C_3_O_4_)_3_]4H_2_O
*M*_r_	757.66
Crystal system, space group	Monoclinic, *C*2/*c*
Temperature (K)	296
*a*, *b*, *c* ()	19.1141(5), 16.7537(4), 11.0053(2)
()	98.803(1)
*V* (^3^)	3482.73(14)
*Z*	4
Radiation type	Mo *K*
(mm^1^)	0.41
Crystal size (mm)	0.58 0.21 0.14

Data collection	
Diffractometer	Bruker APEXII CCD
Absorption correction	Multi-scan (*SADABS*; Krause *et al.*, 2015 ▸)
*T*_min_, *T*_max_	0.708, 0.746
No. of measured, independent and observed [*I* > 2(*I*)] reflections	56955, 5322, 3757
*R*_int_	0.038
(sin /)_max_ (^1^)	0.714

Refinement	
*R*[*F* ^2^ > 2(*F* ^2^)], *wR*(*F* ^2^), *S*	0.038, 0.120, 1.03
No. of reflections	5322
No. of parameters	249
No. of restraints	6
H-atom treatment	H atoms treated by a mixture of independent and constrained refinement
_max_, _min_ (e ^3^)	0.23, 0.42

Computer programs: *SAINT* and *APEX2* (Bruker, 2014 ▸), *SHELXS97* (Sheldrick, 2008 ▸), *SHELXL2014* (Sheldrick, 2015 ▸), *OLEX2* (Dolomanov *et al.*, 2009 ▸), *publCIF* (Westrip, 2010 ▸) and *PLATON* (Spek, 2009 ▸).

## supplementary crystallographic information

### Crystal data

**Table:**

(C_7_H_11_N_2_)_3_[Cr(C_3_O_4_)_3_]·4H_2_O	*F*(000) = 1588
*M**_r_* = 757.66	*D*_x_ = 1.445 Mg m^−^^3^
Monoclinic, *C*2/*c*	Mo *K*α radiation, λ = 0.71073 Å
*a* = 19.1141 (5) Å	Cell parameters from 9987 reflections
*b* = 16.7537 (4) Å	θ = 2.4–27.8°
*c* = 11.0053 (2) Å	µ = 0.41 mm^−^^1^
β = 98.803 (1)°	*T* = 296 K
*V* = 3482.73 (14) Å^3^	Prism, violet
*Z* = 4	0.58 × 0.21 × 0.14 mm

### Data collection

**Table:**

Bruker APEXII CCD diffractometer	3757 reflections with *I* > 2σ(*I*)
Radiation source: sealed X-ray tube	*R*_int_ = 0.038
φ and ω scans	θ_max_ = 30.5°, θ_min_ = 2.6°
Absorption correction: multi-scan (*SADABS*; Krause *et al.*, 2015)	*h* = −27→27
*T*_min_ = 0.708, *T*_max_ = 0.746	*k* = −23→23
56955 measured reflections	*l* = −15→15
5322 independent reflections	

### Refinement

**Table:**

Refinement on *F*^2^	Primary atom site location: structure-invariant direct methods
Least-squares matrix: full	Secondary atom site location: difference Fourier map
*R*[*F*^2^ > 2σ(*F*^2^)] = 0.038	Hydrogen site location: mixed
*wR*(*F*^2^) = 0.120	H atoms treated by a mixture of independent and constrained refinement
*S* = 1.03	*w* = 1/[σ^2^(*F*_o_^2^) + (0.056*P*)^2^ + 1.554*P*] where *P* = (*F*_o_^2^ + 2*F*_c_^2^)/3
5322 reflections	(Δ/σ)_max_ = 0.001
249 parameters	Δρ_max_ = 0.23 e Å^−^^3^
6 restraints	Δρ_min_ = −0.42 e Å^−^^3^

### Special details

**Table:**

Geometry. All e.s.d.'s (except the e.s.d. in the dihedral angle between two l.s. planes) are estimated using the full covariance matrix. The cell e.s.d.'s are taken into account individually in the estimation of e.s.d.'s in distances, angles and torsion angles; correlations between e.s.d.'s in cell parameters are only used when they are defined by crystal symmetry. An approximate (isotropic) treatment of cell e.s.d.'s is used for estimating e.s.d.'s involving l.s. planes.

### Fractional atomic coordinates and isotropic or equivalent isotropic displacement parameters (Å^2^)

**Table:**

	*x*	*y*	*z*	*U*_iso_*/*U*_eq_	
Cr1	0.5000	0.25122 (2)	0.2500	0.04246 (11)	
O2	0.44865 (6)	0.33279 (7)	0.32829 (9)	0.0502 (3)	
O1	0.41659 (6)	0.25871 (7)	0.12056 (10)	0.0521 (3)	
O5	0.45866 (6)	0.16208 (7)	0.33354 (10)	0.0522 (3)	
O7W	0.82663 (8)	0.36686 (8)	0.49953 (15)	0.0698 (4)	
O6	0.45686 (8)	0.02935 (8)	0.34038 (13)	0.0695 (4)	
O3	0.35256 (8)	0.40942 (8)	0.29279 (12)	0.0714 (4)	
N2	0.28924 (8)	0.40160 (8)	0.65828 (12)	0.0507 (3)	
O8W	0.87475 (9)	0.52183 (8)	0.53015 (16)	0.0762 (4)	
N1	0.33363 (7)	0.16521 (9)	0.60672 (13)	0.0499 (3)	
O4	0.31735 (7)	0.32756 (9)	0.06984 (14)	0.0767 (4)	
N3	0.5000	0.87873 (15)	0.2500	0.0615 (5)	
C6	0.30440 (7)	0.32468 (9)	0.64377 (12)	0.0405 (3)	
N4	0.5000	0.63400 (15)	0.2500	0.0730 (7)	
C1	0.39125 (9)	0.35760 (9)	0.26335 (14)	0.0475 (3)	
C7	0.34073 (8)	0.29953 (10)	0.54716 (12)	0.0442 (3)	
H7	0.3556	0.3370	0.4943	0.053*	
C2	0.37147 (9)	0.31285 (10)	0.13931 (15)	0.0496 (4)	
C5	0.28590 (9)	0.26379 (10)	0.72219 (14)	0.0483 (4)	
H5	0.2636	0.2770	0.7888	0.058*	
C3	0.47543 (8)	0.09271 (10)	0.30033 (14)	0.0486 (4)	
C4	0.30043 (9)	0.18699 (11)	0.70089 (14)	0.0521 (4)	
H4	0.2872	0.1478	0.7527	0.063*	
C12	0.5000	0.71391 (15)	0.2500	0.0505 (5)	
C8	0.35377 (8)	0.22127 (10)	0.53192 (14)	0.0476 (3)	
H8	0.3773	0.2057	0.4679	0.057*	
C10	0.30788 (12)	0.46256 (11)	0.57456 (17)	0.0626 (5)	
H10A	0.3583	0.4633	0.5769	0.094*	
H10B	0.2924	0.5138	0.5991	0.094*	
H10C	0.2853	0.4508	0.4925	0.094*	
C13	0.47586 (10)	0.75888 (12)	0.34435 (16)	0.0563 (4)	
H13	0.4593	0.7330	0.4091	0.068*	
C14	0.47685 (9)	0.83913 (12)	0.34033 (17)	0.0608 (4)	
H14	0.4608	0.8678	0.4030	0.073*	
C9	0.25007 (13)	0.42746 (13)	0.75514 (19)	0.0741 (6)	
H9A	0.2037	0.4041	0.7419	0.111*	
H9B	0.2460	0.4846	0.7537	0.111*	
H9C	0.2748	0.4108	0.8336	0.111*	
C11	0.47408 (17)	0.58935 (16)	0.3474 (3)	0.1074 (10)	
H11A	0.5028	0.6012	0.4247	0.161*	
H11B	0.4764	0.5332	0.3310	0.161*	
H11C	0.4259	0.6041	0.3509	0.161*	
H3	0.5000	0.934 (2)	0.2500	0.091 (11)*	
H7WA	0.7843 (7)	0.3544 (19)	0.481 (3)	0.136*	
H8WA	0.8595 (16)	0.4767 (9)	0.528 (3)	0.136*	
H8WB	0.8988 (14)	0.5268 (17)	0.475 (2)	0.136*	
H7WB	0.8432 (14)	0.3280 (13)	0.540 (3)	0.136*	
H1	0.3406 (11)	0.1122 (13)	0.5955 (18)	0.068 (6)*	

### Atomic displacement parameters (Å^2^)

**Table:**

	*U*^11^	*U*^22^	*U*^33^	*U*^12^	*U*^13^	*U*^23^
Cr1	0.04232 (19)	0.0513 (2)	0.03681 (16)	0.000	0.01578 (13)	0.000
O2	0.0557 (6)	0.0565 (7)	0.0411 (5)	0.0033 (5)	0.0164 (5)	−0.0054 (4)
O1	0.0505 (6)	0.0617 (7)	0.0445 (6)	−0.0005 (5)	0.0087 (5)	−0.0094 (5)
O5	0.0572 (7)	0.0538 (6)	0.0530 (6)	−0.0008 (5)	0.0318 (5)	−0.0011 (5)
O7W	0.0775 (9)	0.0497 (7)	0.0849 (10)	−0.0032 (7)	0.0211 (8)	−0.0005 (7)
O6	0.0851 (9)	0.0556 (7)	0.0795 (9)	−0.0022 (7)	0.0499 (8)	0.0054 (6)
O3	0.0916 (10)	0.0639 (8)	0.0653 (8)	0.0297 (7)	0.0330 (7)	0.0100 (6)
N2	0.0646 (8)	0.0478 (7)	0.0431 (6)	−0.0014 (6)	0.0191 (6)	−0.0055 (5)
O8W	0.0914 (11)	0.0531 (8)	0.0954 (11)	−0.0067 (7)	0.0501 (9)	−0.0132 (7)
N1	0.0509 (7)	0.0481 (8)	0.0498 (7)	0.0025 (6)	0.0041 (6)	−0.0044 (6)
O4	0.0598 (8)	0.0853 (10)	0.0794 (9)	0.0068 (7)	−0.0076 (7)	0.0037 (8)
N3	0.0568 (12)	0.0558 (13)	0.0701 (14)	0.000	0.0036 (10)	0.000
C6	0.0399 (7)	0.0492 (8)	0.0323 (6)	−0.0036 (6)	0.0057 (5)	−0.0039 (5)
N4	0.0845 (17)	0.0570 (13)	0.0703 (14)	0.000	−0.0111 (12)	0.000
C1	0.0565 (9)	0.0447 (8)	0.0467 (8)	0.0018 (7)	0.0250 (7)	0.0078 (6)
C7	0.0462 (8)	0.0530 (8)	0.0351 (6)	−0.0040 (6)	0.0123 (6)	−0.0008 (6)
C2	0.0469 (8)	0.0526 (9)	0.0512 (8)	−0.0051 (7)	0.0132 (7)	0.0065 (7)
C5	0.0542 (9)	0.0568 (9)	0.0363 (7)	−0.0040 (7)	0.0150 (6)	−0.0003 (6)
C3	0.0462 (8)	0.0569 (9)	0.0469 (8)	−0.0014 (7)	0.0205 (6)	0.0010 (7)
C4	0.0598 (10)	0.0540 (9)	0.0431 (8)	−0.0056 (7)	0.0098 (7)	0.0052 (7)
C12	0.0461 (12)	0.0573 (14)	0.0448 (11)	0.000	−0.0033 (9)	0.000
C8	0.0435 (8)	0.0603 (9)	0.0401 (7)	0.0017 (7)	0.0097 (6)	−0.0075 (6)
C10	0.0877 (14)	0.0462 (9)	0.0567 (10)	−0.0061 (9)	0.0197 (9)	−0.0016 (7)
C13	0.0509 (9)	0.0763 (13)	0.0430 (8)	−0.0002 (8)	0.0113 (7)	0.0062 (7)
C14	0.0546 (10)	0.0712 (12)	0.0572 (10)	0.0095 (9)	0.0107 (8)	−0.0094 (8)
C9	0.1006 (16)	0.0638 (12)	0.0665 (11)	0.0046 (11)	0.0406 (11)	−0.0154 (9)
C11	0.127 (2)	0.0759 (16)	0.108 (2)	−0.0233 (15)	−0.0179 (17)	0.0327 (14)

### Geometric parameters (Å, º)

**Table:**

Cr1—O2^i^	1.9577 (11)	C6—C5	1.416 (2)
Cr1—O2	1.9577 (11)	N4—C12	1.339 (3)
Cr1—O1^i^	1.9728 (12)	N4—C11	1.455 (3)
Cr1—O1	1.9728 (12)	N4—C11^i^	1.455 (3)
Cr1—O5^i^	1.9804 (11)	C1—C2	1.553 (2)
Cr1—O5	1.9804 (11)	C7—H7	0.9300
O2—C1	1.2834 (19)	C7—C8	1.350 (2)
O1—C2	1.290 (2)	C5—H5	0.9300
O5—C3	1.274 (2)	C5—C4	1.344 (2)
O7W—H7WA	0.830 (10)	C3—C3^i^	1.558 (3)
O7W—H7WB	0.822 (10)	C4—H4	0.9300
O6—C3	1.223 (2)	C12—C13^i^	1.416 (2)
O3—C1	1.2162 (19)	C12—C13	1.416 (2)
N2—C6	1.336 (2)	C8—H8	0.9300
N2—C10	1.456 (2)	C10—H10A	0.9600
N2—C9	1.459 (2)	C10—H10B	0.9600
O8W—H8WA	0.810 (10)	C10—H10C	0.9600
O8W—H8WB	0.818 (10)	C13—H13	0.9300
N1—C4	1.346 (2)	C13—C14	1.345 (3)
N1—C8	1.343 (2)	C14—H14	0.9300
N1—H1	0.91 (2)	C9—H9A	0.9600
O4—C2	1.214 (2)	C9—H9B	0.9600
N3—C14^i^	1.326 (2)	C9—H9C	0.9600
N3—C14	1.326 (2)	C11—H11A	0.9600
N3—H3	0.92 (4)	C11—H11B	0.9600
C6—C7	1.4200 (19)	C11—H11C	0.9600
			
O2^i^—Cr1—O2	91.45 (7)	O4—C2—O1	124.63 (17)
O2—Cr1—O1	82.48 (5)	O4—C2—C1	121.68 (16)
O2—Cr1—O1^i^	92.41 (5)	C6—C5—H5	119.8
O2^i^—Cr1—O1	92.41 (5)	C4—C5—C6	120.39 (15)
O2^i^—Cr1—O1^i^	82.47 (5)	C4—C5—H5	119.8
O2—Cr1—O5^i^	173.35 (5)	O5—C3—C3^i^	114.17 (8)
O2—Cr1—O5	93.41 (5)	O6—C3—O5	126.08 (14)
O2^i^—Cr1—O5	173.35 (5)	O6—C3—C3^i^	119.75 (9)
O2^i^—Cr1—O5^i^	93.41 (5)	N1—C4—H4	119.1
O1—Cr1—O1^i^	172.70 (7)	C5—C4—N1	121.88 (15)
O1—Cr1—O5^i^	92.78 (5)	C5—C4—H4	119.1
O1^i^—Cr1—O5	92.79 (5)	N4—C12—C13	122.15 (11)
O1—Cr1—O5	92.72 (5)	N4—C12—C13^i^	122.15 (11)
O1^i^—Cr1—O5^i^	92.72 (5)	C13—C12—C13^i^	115.7 (2)
O5^i^—Cr1—O5	82.11 (6)	N1—C8—C7	121.82 (14)
C1—O2—Cr1	115.12 (10)	N1—C8—H8	119.1
C2—O1—Cr1	114.55 (10)	C7—C8—H8	119.1
C3—O5—Cr1	114.77 (9)	N2—C10—H10A	109.5
H7WA—O7W—H7WB	102 (2)	N2—C10—H10B	109.5
C6—N2—C10	121.50 (13)	N2—C10—H10C	109.5
C6—N2—C9	121.25 (15)	H10A—C10—H10B	109.5
C10—N2—C9	117.18 (15)	H10A—C10—H10C	109.5
H8WA—O8W—H8WB	108 (2)	H10B—C10—H10C	109.5
C4—N1—H1	117.7 (13)	C12—C13—H13	120.0
C8—N1—C4	119.70 (15)	C14—C13—C12	119.98 (17)
C8—N1—H1	122.5 (13)	C14—C13—H13	120.0
C14—N3—C14^i^	120.0 (3)	N3—C14—C13	122.18 (19)
C14^i^—N3—H3	120.02 (13)	N3—C14—H14	118.9
C14—N3—H3	120.01 (13)	C13—C14—H14	118.9
N2—C6—C7	121.05 (14)	N2—C9—H9A	109.5
N2—C6—C5	122.92 (13)	N2—C9—H9B	109.5
C5—C6—C7	116.04 (14)	N2—C9—H9C	109.5
C12—N4—C11^i^	120.94 (15)	H9A—C9—H9B	109.5
C12—N4—C11	120.93 (15)	H9A—C9—H9C	109.5
C11^i^—N4—C11	118.1 (3)	H9B—C9—H9C	109.5
O2—C1—C2	113.93 (13)	N4—C11—H11A	109.5
O3—C1—O2	125.85 (16)	N4—C11—H11B	109.5
O3—C1—C2	120.19 (15)	N4—C11—H11C	109.5
C6—C7—H7	119.9	H11A—C11—H11B	109.5
C8—C7—C6	120.14 (14)	H11A—C11—H11C	109.5
C8—C7—H7	119.9	H11B—C11—H11C	109.5
O1—C2—C1	113.67 (13)		
			
Cr1—O2—C1—O3	−177.75 (14)	C7—C6—C5—C4	−2.3 (2)
Cr1—O2—C1—C2	4.37 (16)	C5—C6—C7—C8	2.0 (2)
Cr1—O1—C2—O4	178.94 (14)	C4—N1—C8—C7	−1.0 (2)
Cr1—O1—C2—C1	−2.76 (16)	C12—C13—C14—N3	−0.1 (3)
Cr1—O5—C3—O6	179.09 (15)	C8—N1—C4—C5	0.7 (2)
Cr1—O5—C3—C3^i^	−0.6 (2)	C10—N2—C6—C7	1.3 (2)
O2—C1—C2—O1	−1.05 (19)	C10—N2—C6—C5	−178.85 (16)
O2—C1—C2—O4	177.30 (15)	C13^i^—C12—C13—C14	0.02 (12)
O3—C1—C2—O1	−179.06 (15)	C14^i^—N3—C14—C13	0.03 (13)
O3—C1—C2—O4	−0.7 (2)	C9—N2—C6—C7	178.16 (16)
N2—C6—C7—C8	−178.10 (14)	C9—N2—C6—C5	−2.0 (3)
N2—C6—C5—C4	177.81 (15)	C11^i^—N4—C12—C13	−179.27 (16)
C6—C7—C8—N1	−0.4 (2)	C11^i^—N4—C12—C13^i^	0.74 (16)
C6—C5—C4—N1	1.0 (3)	C11—N4—C12—C13^i^	−179.26 (16)
N4—C12—C13—C14	−179.98 (12)	C11—N4—C12—C13	0.74 (16)

Symmetry code: (i) −*x*+1, *y*, −*z*+1/2.

### Hydrogen-bond geometry (Å, º)

**Table:**

*D*—H···*A*	*D*—H	H···*A*	*D*···*A*	*D*—H···*A*
N1—H1···O8*W*^ii^	0.91 (2)	1.84 (2)	2.702 (2)	157.8 (19)
N3—H3···O6^iii^	0.92 (4)	2.12 (3)	2.879 (3)	139 (1)
N3—H3···O6^iv^	0.92 (4)	2.12 (3)	2.879 (3)	139 (1)
O7*W*—H7*WA*···O4^i^	0.83 (1)	1.99 (1)	2.819 (2)	178 (3)
O7*W*—H7*WB*···O1^v^	0.82 (1)	2.12 (1)	2.9079 (19)	161 (3)
O8*W*—H8*WA*···O7*W*	0.81 (1)	1.95 (1)	2.7578 (19)	172 (3)
O8*W*—H8*WB*···O6^vi^	0.82 (1)	1.99 (1)	2.8007 (19)	175 (3)

Symmetry codes: (i) −*x*+1, *y*, −*z*+1/2; (ii) *x*−1/2, *y*−1/2, *z*; (iii) −*x*+1, *y*+1, −*z*+1/2; (iv) *x*, *y*+1, *z*; (v) *x*+1/2, −*y*+1/2, *z*+1/2; (vi) *x*+1/2, *y*+1/2, *z*.

## References

[bb1] Bélombé, M. M., Nenwa, J., Mbiangué, Y. A., Bebga, G., Majoumo-Mbé, F., Hey-Hawkins, E. & Lönnecke, P. (2009*a*). *Inorg. Chim. Acta*, **362**, 1–4. 10.1039/b818793b 19488450

[bb2] Bélombé, M. M., Nenwa, J., Mbiangué, Y. A., Majoumo-Mbé, F., Lönnecke, P. & Hey-Hawkins, E. (2009*b*). *Dalton Trans.* pp. 4519. 10.1039/b818793b 19488450

[bb3] Bélombé, M. M., Nenwa, J., Mbiangué, Y. A., Nnanga, G. E., Mbomékallé, I. M., Hey-Hawkins, E., Lönnecke, P. & Majoumo, F. (2003). *Dalton Trans.* pp. 2117–2118.

[bb4] Ben Nasr, M., Lefebvre, F. & Ben Nasr, C. (2015). *Am. J. Anal. Chem.* **6**, 446–456.

[bb5] Benslimane, M., Merazig, H., Daran, J.-C. & Zeghouan, O. (2012). *Acta Cryst.* E**68**, m1321–m1322. 10.1107/S1600536812040901 PMC3515091 23284318

[bb6] Bruker (2014). *APEX2* and *SAINT*. Bruker AXS Inc., Madison, Wisconsin, USA.

[bb7] Dolomanov, O. V., Bourhis, L. J., Gildea, R. J., Howard, J. A. K. & Puschmann, H. (2009). *J. Appl. Cryst.* **42**, 339–341.

[bb8] Ghouili, A., Chaari, N. & Zouari, F. (2010). *X-ray Struct. Anal. Online*, **26**, 21–22.

[bb9] Golič, L. & Bulc, N. (1988). *Acta Cryst.* C**44**, 2065–2068.

[bb10] Krause, L., Herbst-Irmer, R., Sheldrick, G. M. & Stalke, D. (2015). *J. Appl. Cryst.* **48**, 3–10. 10.1107/S1600576714022985 PMC4453166 26089746

[bb11] Martin, L., Day, P., Clegg, W., Harrington, R. W., Horton, P. N., Bingham, A., Hursthouse, M. B., McMillan, P. & Firth, S. (2007). *J. Mater. Chem.* **17**, 3324–3329.

[bb12] Mbiangué, Y. A., Nenwa, J., Bélombé, M. M., Ngoune, J. & Álvarez, E. (2012). *ScienceJet*, **1**, 1–9.

[bb13] Nenwa, J., Belombe, M. M., Ngoune, J. & Fokwa, B. P. T. (2010). *Acta Cryst.* E**66**, m1410. 10.1107/S1600536810040353 PMC3009100 21588840

[bb14] Neo, K. E., Ong, Y. Y., Huynh, H. V. & Andy-Hor, T. S. (2006). *J. Mater. Chem.* **17**, 1002–1006.

[bb15] Sheldrick, G. M. (2008). *Acta Cryst.* A**64**, 112–122. 10.1107/S0108767307043930 18156677

[bb16] Sheldrick, G. M. (2015). *Acta Cryst.* C**71**, 3–8.

[bb17] Spek, A. L. (2009). *Acta Cryst.* D**65**, 148–155. 10.1107/S090744490804362X PMC2631630 19171970

[bb18] Westrip, S. P. (2010). *J. Appl. Cryst.* **43**, 920–925.

